# Comments on “Implications of portal vein/superior mesenteric vein involvement in pancreatic cancer: a comprehensive correlation from preoperative radiological assessment to resection, pathology, and long-term outcomes. a retrospective cohort study”

**DOI:** 10.1097/JS9.0000000000002650

**Published:** 2025-06-05

**Authors:** Miao Qiu, Zhongkui Xiong, Mengyu Lyu

**Affiliations:** a Department of Tumor Radiotherapy, Shaoxing Second Hospital, Shaoxing, Zhejiang, China; b Pharmacy Department, Shaoxing Second Hospital, Shaoxing, Zhejiang, China

*Dear Editor*,

We read with great interest the recent article entitled “Implications of portal vein/superior mesenteric vein involvement in pancreatic cancer: A comprehensive correlation from preoperative radiological assessment to resection, pathology, and long-term outcomes. A retrospective cohort study” published in *Int J Surg*. While commending the authors’ efforts to address this surgically relevant topic, we wish to highlight several methodological and interpretative concerns that warrant clarification to ensure scholarly accuracy^[1]^.

The statement *”We analyzed 443 patients undergoing pancreatoduodenectomy at a tertiary center from 2012 to 2017 based on PV/SMV resection”* could be misinterpreted as implying universal PV/SMV resection among all 443 patients. However, subsequent analyses clearly stratify outcomes by resection status (e.g., PV/SMV resection (+) vs. PV/SMV resection (-)). To prevent reader confusion, we recommend revising this to: *”We analyzed 443 patients undergoing pancreatoduodenectomy from 2012 to 2017, stratified by PV/SMV resection status .”*

The reported survival comparison states: *”Patients without PV/SMV resection with an rR1/R1 margin showed no decrease in survival compared to those with PV/SMV resection and R0 margins (54.9% vs 40.3%, *P* = 0.029).”* This interpretation appears discordant with both the pre-specified significance threshold (*α* = 0.05) and conventional statistical norms. A *P*-value of 0.029 indicates a statistically significant difference, directly contradicting the “no decrease” conclusion. This discrepancy necessitates re-examination of either the statistical methodology or the interpretative framework.

The author included preoperative neoadjuvant therapy as the research content, but only chemotherapy as neoadjuvant therapy and did not include preoperative radiotherapy. In fact, neoadjuvant therapy includes preoperative radiotherapy. This weakens the comprehensiveness of the research and may lead to biased research results.

We appreciate the efforts made by Kim *et al*^[2]^. Though this study provides valuable insights into PV/SMV management strategies, the identified issues – ambiguous cohort description, paradoxical statistical interpretation, and incomplete therapeutic scope – may inadvertently mislead clinical readers. Addressing these concerns through transparent data re-analysis and methodological clarification would significantly strengthen the manuscript’s scientific rigor and clinical applicability.

## Data Availability

Not applicable.
